# An exploratory analysis of the impact of a university campus smoking ban on staff and student smoking habits in Japan

**DOI:** 10.1186/1617-9625-11-19

**Published:** 2013-09-15

**Authors:** Hiroki Ohmi, Toshiyuki Okizaki, Martin Meadows, Kazuyuki Terayama, Yoshikatsu Mochizuki

**Affiliations:** 1 Department of Nutritional Sciences, Faculty of Health and Welfare Science, Nayoro City University, W4-N8, Nayoro 096-8641, Hokkaido, Japan; 2 Department of Liberal Arts Education, Faculty of Health and Welfare Science, Nayoro City University, W4-N8, Nayoro 096-8641, Hokkaido, Japan; 3 School of Nursing Science, Asahikawa Medical University, E2-1-1-1, Midorigaoka, Asahikawa 078-8510, Japan

**Keywords:** University smoking policy, Total smoking ban, Impact on smokers

## Abstract

**Background:**

Smoking bans in public places have been shown to have an impact on smoking habits, however the potential influence of a university smoking ban on faculty and staff smoking habits remains elusive.

**Methods:**

This cross sectional study was implemented in Nayoro City, Japan in 2011, among the faculty and students of the Nayoro City University. Five years after the declaration of a total ban on smoking on a university campus, the smoking characteristics of all students, teachers and office workers, and the policy’s impact on smokers were investigated. The survey was conducted through an anonymous, self-administered, multiple-choice questionnaire. Information was gathered on the characteristics and smoking characteristics of respondents, and the smokers attitudes toward smoking.

**Results:**

The recovery rate was 62.1%. Among respondents, smoking prevalence was 17.9% in teachers and office workers, and 4.0% in students. Among all smokers, 46.4% did not abstain from smoking while at the university and they indicated their smoking areas were “on the streets next to the campus”: 16 and “outdoors on campus”: 3, respectively. As for smokers, 29.6% of them reduced the number of cigarettes smoked per day as a result of the smoking ban. None of the ex-smokers replied that their principal motivation for quitting smoking was the smoking ban.

**Conclusions:**

The ban on smoking served a motivator for smokers to reduce in smoking, but not serve as an effective motivator to quit smoking.

## Background

In Japan, a health promotion plan named “Healthy Japan 21” was published by the Ministry of Health and Welfare in 2000 [1]. In the section dealing with anti-tobacco measures, four aims were set. One of these aims was to protect non-smokers from second-hand smoke in public spaces. After release of the plan, several institutions endeavored to protect their users from second-hand smoke, however they failed to take sufficient measures. In order to give legal infrastructure to the plan, the Health Promotion Law was implemented in 2003. According to this law, it is the duty of institutional managers to provide protection from passive smoking. Article 25 of the law states that “persons in charge of management at facilities used by large numbers of people, such as schools, gymnasiums, hospitals, theaters, viewing stands, assembly halls, exhibition halls, department stores, offices, public facilities, and eating and drinking places shall endeavor to take necessary measures to protect users of these facilities from being exposed to second-hand smoke, (passive smoking refers to being forced to inhale other people’s second hand smoke in an indoor or equivalent environment)” [2].

A number of universities and colleges have declared a smoke-free policy on campus, based on this law [3]. Recent decreases in the prevalence of smoking among Japanese adolescents may be due partly to such smoke-free policies at school [4]. However, the impact of a school’s smoke-free policy on the entire population of students, teachers and office workers who habitually smoke has not been conclusively identified.

In Nayoro City University, a smoking policy that includes a total ban on smoking both indoors and outdoors on campus was declared in 2006. Five years after introduction, the smoking characteristics of the entire university population and the impact of the total smoking ban on smokers were investigated in 2011.

## Methods

### Subjects

In May 2011, Nayoro City University was comprised of 101 teachers and office workers (male: 50, female: 51) and 712 students (male: 118, female: 594), all of whom participated in the survey. Students belonged to The Faculty of Health and Welfare Science or Early Childhood Education.

### Data collection

Data collection was done anonymously in May 2011 by means of a self-administered, multiple-choice questionnaire. The questionnaire asked for information about gender, status in the school (i.e. teacher, office worker or student), and smoking characteristics (i.e. non-smoker, ex-smoker, or smoker). Ex-smokers were asked about their motivations for quitting smoking. The questionnaire also asked smokers about the number of cigarettes smoked per day, years of smoking, items from the Fagerström Test for Nicotine Dependence (FTND), their smoking habits while at the university (i.e. abstinence or not), smoking area while at the university, impact of the smoking ban, the effect of a price increase on the number of cigarettes smoked per day, and their intention to quit smoking.

The protocol of this study was approved by the Ethics Committee of Nayoro City University.

### Statistical analysis

Data were digitized then analyzed with descriptive statistics according to characteristics of subjects. With respect to smokers, differences in years of smoking and FTND between teachers/office workers, and students were analyzed by Student’s *t*-test and Mann Whitney *U*-test, respectively. In the same way, the relationship between smoking habits of smokers while at the university, and smoking characteristics and FTND were analyzed with the same tests. All *P*-values were based on a two-tailed test and a significance level lower than 0.05 was defined as significant. Statistical analysis was performed using the Dr. SPSS 2 for Windows 11.0.1 J statistical package.

## Results

### Smoking prevalence

Among 813 subjects, we obtained valid responses from 56 teachers and office workers (recovery rate: 55.4%) and 449 students (recovery rate: 63.1%) (Table 1). Among respondents, the smoking prevalence was 17.9% among teachers and office workers, and 4.0% among students.

**Table 1:** Smoking prevalence of staff and students of the Nayaro City University, Japan, 2011

		**Subject**	**Respondent**		**n (%)**	
				**Non-smoker**	**Ex-smoker**	**Smoker**
Teachers & office workers	Male	50	25	13 (52.0)	7 (28.0)	5 (20.0)
	Female	51	31	18 (58.1)	8 (25.8)	5 (16.1)
	Subtotal	101	56	31 (55.4)	15 (26.8)	10 (17.9)
Students	Male	118	68	59 (86.8)	3 (4.4)	6 (8.8)
	Female	594	381	362 (95.0)	7 (1.8)	12 (3.1)
	Subtotal	712	449	421 (93.8)	10 (2.2)	18 (4.0)
Total		813	505	452 (89.5)	25 (5.0)	28 (5.5)

### Motivators for ex-smokers to quit smoking

Ex-smokers were asked to indicate the most appropriate motivator THAT LEAD THEM TO QUIT SMOKING. Among the listed motivators, none of the ex-smokers replied that the most appropriate motivator for quitting smoking was the ban on smoking (Table 2).

**Table 2:** Motivators for ex-smokers to quit smoking of staff and students of the Nayaro City University, Japan, 2011

**Most appropriate motivator**	**Teachers &office workers**		**Students**		**Subtotal (%)**
	**Male**	**Female**	**Male**	**Female**	
Ban on smoking	0	0	0	0	0 ( 0.0)
Price increase	2	1	1	1	5 (23.8)
Own health	2	2	1	4	9 (42.9)
Partner’s health	1	1	0	0	2 ( 9.5)
Child’s health	0	1	0	0	1 ( 4.8)
Partner’s comfort	0	0	0	2	2 ( 9.5)
Other	1	1	0	0	2 ( 9.5)

### Smoking characteristics and habits of smokers

The smoking characteristics of smokers is shown in Table 3. The number of years smoking among teachers and office workers was greater than among students (*P*=0.007: Student’s *t*-test). Statistically, there was no difference in FTND between teachers/office workers, and students (Mann Whitney *U*-test). Among all smokers, 46.4% did not abstain from smoking while at the university and they indicated their smoking areas were “on the streets next to the campus”: 16 and “outdoors on campus”: 3, respectively (Table 4). Number of cigarettes smoked per day and FTND among non-abstinent smokers were significantly greater than those among the abstinent smokers (number of cigarette: *P*=0.001: Student’s *t*-test and FTND:*P*=0.029: Mann Whitney *U*-test, respectively, Table 5).

**Table 3:** Smoking characteristics of smokers of staff and students of the Nayaro City University, Japan, 2011

		**n**	**mean**		**SD**
Number of cigarettes smoked per day					
Teachers & office workers	male	5	15.4		5.3
	female	4	12.5		6.5
Students	male	6	10.7		4.8
	female	12	11.8		6.6
Number of years of smoking		n	mean		SD
Teachers & office workers	male	5	22.2		10.1
	female	4	11.5		10.5
Students	male	6	4.7		3.8
	female	12	4.0		1.6
			*P*=0.007: Student’s *t*-test		
FTND		n	median	min	max
Teachers & office workers	male	5	5	3	8
	female	4	1.5	0	3
Students	male	6	2	1	5
	female	12	2	0	6

*P*=ns, Mann Whitney *U*-test.

*FTND* Fagerström test for nicotine dependence.

**Table 4:** Smoking habits while at the university of staff and students of the Nayaro City University, Japan, 2011

	**n (%)**	**Smoking area (multiple answers allowed)**	**n**
Abstinent	15 (53.6)		
Not abstinent	13 (46.4)	On the streets next to the campus	16
		Outdoors on campus	3
		Indoors on campus	0

**Table 5:** Smoking habits while at the university and smoking characteristics of staff and students of the Nayaro City University, Japan, 2011

**Number of cigarettes smoked per day**	**n**	**mean**	**SD**	
Abstinent	13	8.9	3.4	
Not abstinent	14	15.6	5.9	
		*P*=0.001: Student’s *t*-test		
FTND	n	median	min	max
Abstinent	13	2	0	4
Not abstinent	14	3	1	8
		*P*=0.029: Mann Whitney *U*-test		

### Impact of ban on smoking and intention to quit smoking

The impacts of the ban on smoking and the price increase on the number of cigarettes smoked per day are shown in Table 6. As for the smokers’ intention to quit smoking, 48.1% replied “I do not want to quit smoking now”, and another 48.1% replied “I want to quit smoking in the near future”, while only one student replied, “I am trying to quit smoking now” (Table 7).

**Table 6:** Impact of ban on smoking and price increase on number of cigarettes smoked per day of staff and students of the Nayaro City University, Japan, 2011

		**n (%)**
Ban on smoking	Intend to decrease	8 (29.6)
	No intent to decrease	19 (70.4)
Price increase	Intend to decrease	5 (18.5)
	No intent to decrease	22 (81.5)

**Table 7:** Intention to quit smoking of staff and students of the Nayaro City University, Japan, 2011

	**n**		**n (%)**
	**Teachers &office workers**	**Students**	**Subtotal**
I do not want to quit smoking now.	8	5	13 (48.1)
I want to quit smoking in near future.	2	11	13 (48.1)
I want to quit smoking right now.	0	0	0 (0.0)
I am trying to quit smoking now.	0	1	1 (3.8)

## Discussion

### Main findings of this study

According to a previous survey on female students in the Department of Nursing Science at Nayoro City University (students in other departments were not included), smoking prevalence was 12.3% in 2002 and 10.5% in 2003, respectively [5]. In this 2011 survey, smoking prevalence had decreased to 4.3% of students. A school policy restricting smoking based on the Health Promotion Law might be a contributory factor in discouraging the initiation of smoking. As a result, the yearly admission of students who do not later take up smoking may have had a cumulative effect, leading to an overall decrease in the prevalence of smoking [4].

As for smokers, 29.6% of them reduced the number of cigarettes smoked per day as a result of the smoking ban. More than half of smokers abstained from smoking at the university. The ban on smoking was demonstrated to have a certain effect in smoking reduction for smokers.

By contrast, none of the ex-smokers also replied that their principal motivation for quitting smoking was the smoking ban. In the case of smokers, around 30% of them reduced the number of cigarettes smoked per day in consequence of the ban on smoking, however, almost all of them were not willing to quit smoking. The ban on smoking served a motivator for smokers to reduce in smoking, but not serve as an effective motivator to quit smoking.

The preventative effects of a reduction in smoking on smokers’ own tobacco-related diseases differs across studies [6-9]. Although it is still uncertain if a reduction in smoking has any preventative effect on smokers’ tobacco-related diseases, several studies have confirmed the health benefits of quitting smoking [10]. However, almost all the surveyed smokers were unwilling to quit smoking right now in spite of current school policy. For smokers, the ban on smoking did not provide an effective motivator to quit smoking but led to smoking on the streets off campus while at the university. In this study, a heavy addiction was related to non-abstinence while at the university, and smoking on the street. The impact of a smoke-free policy on attitudes and quitting behaviors varies among studies [11-15]. The reason might be that the principal aim of the Health Promotion Law and school policy based on that law is to protect non-smokers from second-hand smoke, but not to encourage smokers to quit smoking. Changing attitudes and behaviors among smokers would be an unintended consequence of this legislation and/or policy. In order to encourage addicted smokers to quit smoking, other approaches that incorporate effective educational and medical measures may be necessary. In Japan, smoking cessation therapy with nicotine gum/patches and varenicline has been covered by health insurance under certain conditions since 2006 [16]. But almost all student smokers are excluded from this insured treatment because of the short duration of their smoking. Smoking cessation therapies for adolescents and young adults with a short history of smoking should be covered by health insurance in order to prevent heavier addiction. In this study, smoking teachers and office workers with a longer smoking history were not any more willing to quit smoking than student smokers.

### Limitations of this study

This study was conducted in a small university with a small sample size. Student subjects constituted a near homogeneous age group. Smoking prevalence was investigated once in 2011 in a cross-sectional survey, but a survey was not conducted in 2006 when the policy was first declared. This study focused on smokers’ attitudes and desires to quit, however non-smokers’ perceptions were not investigated. Although the results may not be generalizable, they do, however, provide some evidence of the impact of a school, smoke-free policy based on the Japanese Health Promotion Law on staff and students who are habitual smokers.

## Conclusions

Five years after the introduction of a university smoke-free policy with a campus-wide smoking ban, the impact of the ban on smokers was investigated. A total ban on smoking served a motivator for smokers to reduce in smoking, but not serve as an effective motivator to quit smoking.

In order to encourage addicted smokers to quit smoking, approaches other than a smoking ban that provide effective educational and medical measures may be crucial.

## Competing interests

All the authors have no competing interest relevant to this article.

## Authors’ contributions

HO had primary responsibility for protocol development, data collection, outcome assessment, preliminary data analysis, and writing of the manuscript. TO participated in the data collection, analysis and contributed to the writing of the manuscript. MM, KT and YM supervised the study, performed the final data analysis, and contributed to the writing of the manuscript. All authors read and approved the final manuscript.
